# TAGINE: fast taxonomy-based feature engineering for microbiome analysis

**DOI:** 10.1093/bioadv/vbag056

**Published:** 2026-02-17

**Authors:** Shiri Baum, Ido Meshulam, Yadid M Algavi, Omri Peleg, Elhanan Borenstein

**Affiliations:** Blavatnik School of Computer Science and AI, Tel Aviv University, Tel Aviv 6997801, Israel; Blavatnik School of Computer Science and AI, Tel Aviv University, Tel Aviv 6997801, Israel; Gray Faculty of Medical and Health Sciences, Tel Aviv University, Tel Aviv 6997801, Israel; Blavatnik School of Computer Science and AI, Tel Aviv University, Tel Aviv 6997801, Israel; Blavatnik School of Computer Science and AI, Tel Aviv University, Tel Aviv 6997801, Israel; Gray Faculty of Medical and Health Sciences, Tel Aviv University, Tel Aviv 6997801, Israel; Santa Fe Institute, Santa Fe, NM 87501, United States

## Abstract

**Summary:**

TAGINE is a feature engineering algorithm that leverages the microbial taxonomic tree to optimize feature sets in microbiome data for predictive modeling. The algorithm starts with features at high taxonomic levels and iteratively splits them into lower-level clades in cases where it improves predictive accuracy, ultimately producing a feature set spanning multiple taxonomic levels. This approach aims to markedly reduce the number of features while preserving biological relevance and interpretability. We compare TAGINE’s performance to other standard and taxonomy-based feature engineering methods on several different datasets, and show that TAGINE yields more compact feature sets and is orders of magnitude faster than other methods, while maintaining predictive accuracy.

**Availability and implementation:**

TAGINE is freely available under the MIT license with source code available at https://github.com/borenstein-lab/tagine_fe.

## 1. Introduction

Recent advances in sequencing technologies have facilitated the high-throughput and high-resolution acquisition of biomedical data (Soon *et al.* 2013). These developments offer tremendous promise for improving disease diagnosis and treatment (Schupack *et al.* 2022), but also introduce considerable analytical, computational, and conceptual challenges. Perhaps the most daunting challenge when analyzing such high-dimensional data, where the number of features often vastly exceeds the number of samples (Bellman and Kalaba 1959), is the risk of identifying spurious associations or overfitting the predictive model to the training data. Traditional feature selection and engineering methods aim to mitigate this issue by pinpointing highly informative features or by combining multiple features into composite ones using a variety of statistical or computational approaches (Kuzudisli *et al.* 2023). While these methods provide an effective strategy for many analytical tasks (including the construction of machine-learning predictive models), they often overlook existing biological knowledge, thus limiting model interpretability, failing to utilize available information, and reducing their utility for hypothesis generation.

One notable domain that is characterized by both high-dimensional data and extensive prior biological knowledge is microbiome research. Metagenomics-based profiling of various microbiomes often generates datasets comprising thousands to tens of thousands of species-level taxonomic features (with most studies including only a few dozen to several hundred samples). On the other hand, taxonomic classification systems and phylogenetic trees that link the various taxa offer a natural way to group different taxonomic features. Moreover, since phylogenetic proximity often reflects functional or ecological similarity, such phylogeny- or taxonomy-based grouping is likely analytically and biologically relevant (Goberna and Verdu 2018).

With that in mind, many microbiome studies indeed aggregate features at some coarse taxonomic level (e.g. genus or phylum) prior to downstream analyses in the hope of reducing the number of taxonomic features and improving statistical power (Kleine Bardenhorst *et al.* 2021). However, such fixed-level aggregation may obscure clade-specific differences in granularity and information content, potentially overlooking highly informative features that reside at different taxonomic levels. To address this limitation, a small number of recent methods have introduced tree-guided adaptive grouping of features, aiming to combine informative fine-grained taxonomic features in some clades with coarser-grained features in others. Existing methods target diverse downstream tasks, from dimensionality reduction (Li *et al.* 2022) to regression (Bien *et al.* 2021). Other methods focus mainly, as do we, on classification tasks: HFE (Hierarchical Feature Engineering) (Oudah and Henschel 2018) iteratively identifies informative features along paths in the taxonomic tree, ultimately providing a set of features at various taxonomic levels along these paths, while TaxaHFE (Oliver *et al.* 2023) utilizes an iterative machine learning approach to collapse information-poor features into higher levels and supports both regression and classification tasks. These methods were shown to exhibit impressive predictive performances and feature reduction, while preserving relevant community structure. Importantly, however, these methods are computationally heavy and suffer from relatively slow runtimes, which markedly limit their applicability in large-scale settings. Furthermore, some of these methods can also include multiple features on the same tree path in the final feature set, limiting interpretability.

Motivated by these observations, we introduce TAGINE (Taxonomy-Aware Grouping for INformation Enhancement), a new and fast feature engineering algorithm that leverages the taxonomic tree hierarchy to guide dimensionality reduction for classification tasks. TAGINE begins with coarse-grained taxonomic features and iteratively splits them into finer-grained levels, prioritizing splits that improve predictive accuracy. The resulting grouped features can then be used as inputs for downstream analysis or predictive modeling. To benchmark TAGINE’s effectiveness, we compared its performance to recursive feature elimination (RFE) (Guyon *et al.* 2002), a commonly used, domain-agnostic feature selection algorithm, as well as two of the previously noted taxonomy-based hierarchical feature engineering algorithms. We evaluated the predictive accuracy obtained when using the resulting feature sets, the sizes of these sets, and the runtimes of each method on several independent datasets, showing that TAGINE significantly reduces the number of features and improves runtime while maintaining predictive accuracy.

## 2. The TAGINE algorithm

The TAGINE algorithm takes as input a dataset of taxonomic abundance values, along with a corresponding taxonomic tree structure *T*, such that each feature in the dataset corresponds to a leaf in *T*. Importantly, TAGINE does not assume that the input taxonomic features are at any particular taxonomic level, nor does it require that all features be of the same taxonomic resolution. Internal nodes of *T* represent higher-level taxonomic features, with their corresponding abundance values computed as the sum of their children’s abundances. TAGINE further defines a queue, *Q*, which contains nodes that are candidates for expansion and that will be evaluated iteratively by the algorithm.

TAGINE begins with the tree collapsed up to a predefined taxonomic level (by default, one level below the root), leaving a small number of coarse-grained features, which are subsequently added to *Q*. TAGINE then iteratively pops a collapsed node from *Q*, and compares two models to determine whether this node should be expanded: one that treats the node as a single feature to predict the label, and the other that uses the node’s children as separate features. Specifically, a logistic regression is fitted for each of the two cases, and model fit is then determined using the Akaike Information Criterion (AIC; Akaike 1974). AIC accounts for the different number of parameters in each model, avoiding biasing the algorithm towards expanding nodes with more children. If the AIC suggests that the single-parameter model is a better fit, this means that expanding the node does not improve prediction, and the node remains collapsed as a single feature in the final feature set (which also means its descendants will not be examined by the algorithm). Otherwise, the node is expanded, and any child that represents a collapsed node is added to *Q* for further evaluation. In the implementation analyzed below, to further reduce the number of features, features whose coefficient in the logistic regression was not significant were pruned from *T* (and not added to *Q*).

The algorithm ends when the queue is empty, and the features that remain in *T* as leaves (which may include features at various taxonomic levels, including collapsed nodes) are used as the final feature set.

## 3. Evaluation and results

To evaluate the performance of TAGINE, we applied it to five shotgun metagenomic datasets from the Microbiome-Metabolome curated resource (Muller *et al.* 2022). The datasets included studies of inflammatory bowel disease (IBD; Franzosa *et al.* 2019, IBD (HMP); Lloyd-Price *et al.* 2019), colorectal cancer (CRC; Yachida *et al.* 2019), end-stage renal disease (ESRD; Wang *et al.* 2020), and gastric cancer (GC; Erawijantari *et al.* 2020). In the IBD (HMP) dataset, which is longitudinal, only the first sample from each participant was used. For each dataset, we randomly partitioned the data into 50 training and test sets using an 85%–15% split. We then removed very rare species from each dataset (mean abundance below 10^−6^ or mean prevalence below 10^−2^ in the training set), leaving a mean of 6943.5, 7081.4, 6824.0, 7121.5, and 5059.8 species-level features in the CRC, ESRD, GC, IBD, and IBD (HMP) datasets, respectively. After filtration, the data were normalized to represent relative abundances. Finally, to address zero values, a small constant equal to half the minimum value in each sample was added to all features in that sample, followed by renormalization.

Given these data, we applied TAGINE to each training set (recording the runtime and the resulting number of features), used the selected features to train a random forest model on a classification task, applied this classifier to the test samples, and computed the area under the Receiver Operating Characteristic curve (AUC-ROC; Bradley 1997). As a classification task, we utilized the ESRD and GC datasets in their original case-control format (i.e. classifying cases versus controls). For the CRC dataset, we classified advanced-stage CRC versus all other categories, and for both IBD datasets, we classified UC/CD versus controls.

We compared TAGINE to four other feature selection/engineering methods:

I. No feature selection.
II. Scikit-learn’s (Pedregosa *et al.* 2011) recursive feature elimination algorithm with the commonly used Support Vector Regression estimator, applied to the data after all possible taxonomic levels are included and configured to select the same number of features as TAGINE.
III. HFE (Oudah and Henschel 2018).
IV. TaxaHFE (Oliver *et al.* 2023).

Notably, HFE could not run on the IBD (HMP) dataset as it consistently filtered out all features, and TaxaHFE was used with the super filter feature, as it provided fewer features and better predictive performance.

**Figure 1:**
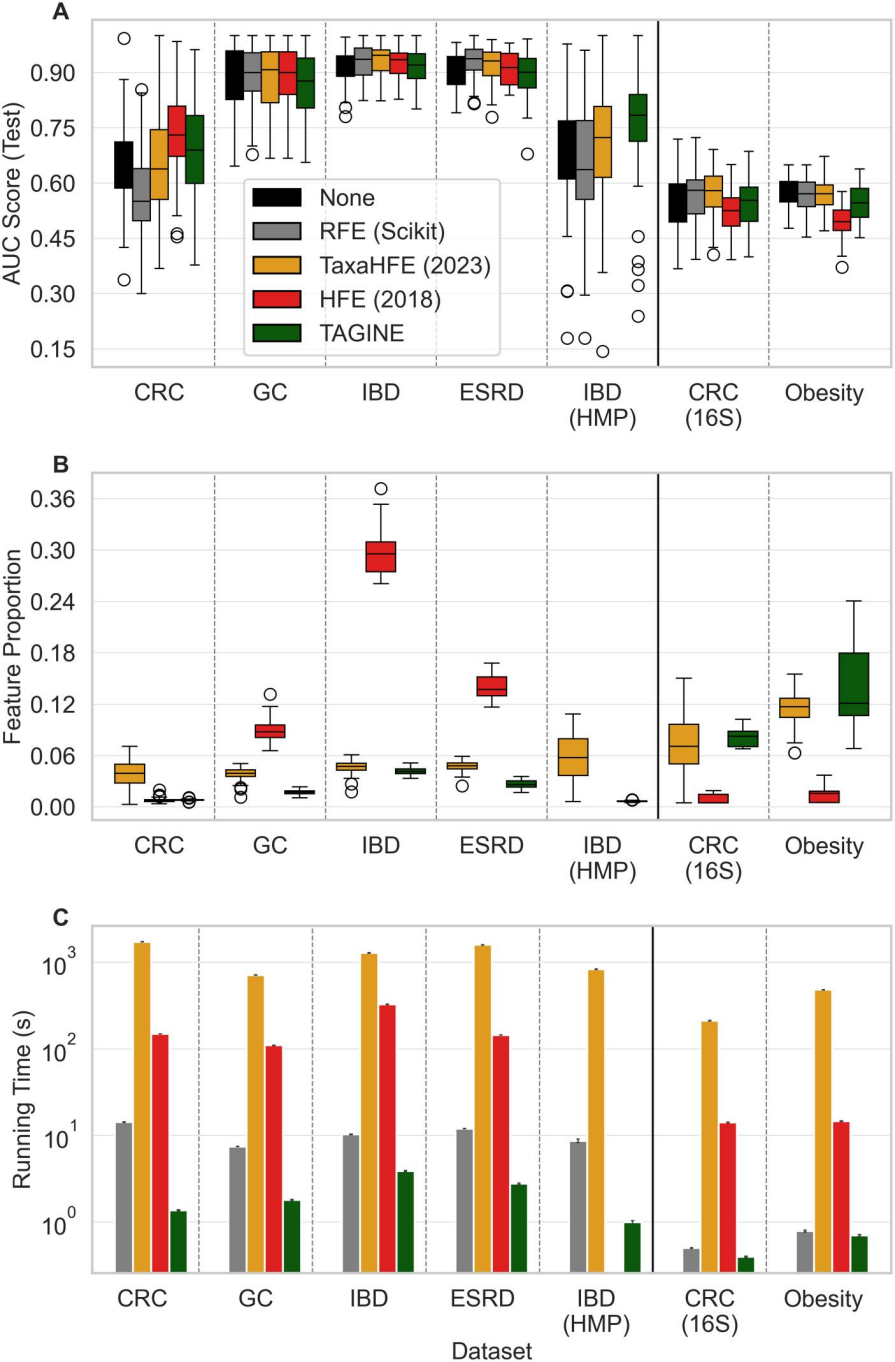
(A) Comparison of AUC scores by dataset and selection method. (B) Comparison of the proportion of features selected out of the original set of features by dataset and selection method. (C) Comparison of running time by dataset and selection method.

We first examined the predictive accuracy obtained with the set of features selected by each approach (Figure 1A; Supplementary Table 1). Significance was evaluated using a Nadeau–Bengio-corrected two-sided t-test over the 50 train-test splits, with Benjamini–Hochberg FDR correction applied for all tests performed on each dataset (Benjamini and Hochberg 1995, Nadeau and Bengio 2003). Overall, all methods exhibited comparable performance across datasets, with no single method significantly outperforming any other method in terms of accuracy on any dataset (Supplementary Table 2). Moreover, even the modest, non-significant differences observed in prediction accuracy were not consistent across datasets, with different methods achieving the highest accuracy in different datasets (e.g. HFE in the CRC dataset, RFE in the ESRD dataset, and TAGINE in the IBD (HMP) datasets).

We then turned to evaluating the number of features selected by each method (Figure 1B; Supplementary Table 1). For this analysis, we naturally only compared TAGINE to HFE and TaxaHFE, since RFE was preconfigured to select the same number of features as TAGINE. We found that in terms of number of features, TAGINE significantly outperformed both tested methods in all shotgun metagenomic datasets in almost all cases (with the only two exceptions being the difference between TAGINE and HFE in the CRC dataset and the difference between TAGINE and TaxaHFE in the IBD dataset, which were not significant; Supplementary Table 2). It is also worth noting the incredibly effective reduction in the number of features selected by TAGINE, with the size of the final feature set being only 0.8%, 2.7%, 1.7%, 4.1%, and 0.7% of the total number of features in the CRC, ESRD, GC, IBD, and IBD (HMP) datasets, respectively. This is particularly impressive given our finding above that the predictive accuracy achieved using these feature sets was comparable to the accuracy achieved using the full set of features.

Importantly, the same trend of comparable predictive accuracy with significantly fewer features holds when comparing TAGINE to simple baseline approaches, either using all features across *all* taxonomic levels (rather than only the finest-grained level present in the original feature set), or using all features at a fixed coarse-grained taxonomic level (Supplementary Figure 1).

To further confirm that TAGINE ultimately selects features from multiple taxonomic levels, we examined the distribution of selected features across taxonomic levels (Supplementary Figure 2). As perhaps expected, the majority of features selected by TAGINE as informative are drawn from the finest available level (species in shotgun metagenomic datasets and genus in 16S rRNA datasets). Evidently, however, TAGINE also consistently selects a substantial proportion of features from coarser taxonomic levels, highlighting its ability to adaptively integrate information across the taxonomic hierarchy. Moreover, when comparing these distributions with TAGINE’s predictive performance, TAGINE appears to perform particularly well in datasets in which the predictive signal is more concentrated at higher taxonomic levels.

Perhaps the biggest advantage of TAGINE is its simplicity and, as a result, its short running time. Indeed, comparing TAGINE to all other methods (except for the no-feature-selection baseline), we found that it was significantly and substantially faster than all other methods in all datasets (Figure 1C; Supplementary Tables 1 and 2). This stems from TAGINE’s algorithmic approach, which performs a single, simple statistical test on a limited number of features per tested node, never fitting a model to the entire feature set and without revisiting the children of nodes that were not expanded. Moreover, as TAGINE examines each inner node in the tree at most once, and since the number of inner nodes in the tree scales linearly with the number of features, TAGINE’s running time scales linearly with the number of features, making this advantage even more pronounced for datasets with a high number of features.

For example, in one of the smaller datasets (GC, with a mean of 6824.0 initial features), TAGINE ran, on average, 4.2 times faster than RFE, 61.1 times faster than HFE, and 395.4 times faster than TaxaHFE (average runtimes 1.8 s for TAGINE, 7.5 s for RFE, 109.9 s for HFE, and 711.7 s for TaxaHFE, measured on a 13th Gen Intel^®^ Core™ i7 processor). As expected, this advantage persisted in larger datasets, with TAGINE running 2.7, 85.8, and 337.3 times faster than RFE, HFE, and TaxaHFE, respectively, on the largest IBD dataset (average runtimes 3.9 s for TAGINE, 10.3 s for RFE, 326.0 s for HFE, and 1281.8 s for TaxaHFE). This reduction in runtime is of great practical relevance, as it makes TAGINE a viable option for large-scale datasets or for tasks requiring multiple iterations, where other methods may prove too computationally demanding to apply.

Finally, to evaluate TAGINE’s performance on substantially smaller datasets, we applied it to two 16S rRNA cohorts from the Microbiome HD collection (Duvallet *et al.* 2017), including a study of CRC (Baxter *et al.* 2016) and obesity (Goodrich *et al.* 2014). The data were processed as described above, and the same feature selection and engineering methods were evaluated. Notably, the two datasets are characterized by a much smaller initial number of features (a mean of 205.0 for CRC, and 189.1 for obesity). On these datasets, TAGINE still significantly outperformed all other methods in terms of runtime in almost all cases (Supplementary Table 2), but was often outperformed in terms of number of features and predictive performance by some other methods, suggesting that TAGINE’s primary advantage is more pronounced on larger datasets.

## 4. Conclusion

We present TAGINE, a fast, taxonomy-based feature engineering algorithm that substantially outperforms existing methods in terms of speed and the number of features selected, while maintaining similar predictive performance. Across the datasets considered, performance varied between methods, with no single approach consistently outperforming the others. In this context, TAGINE’s computational efficiency makes it particularly well suited for scenarios that require repeated feature selection on large, high-dimensional datasets with many features. Naturally, these are precisely the datasets where feature selection and engineering methods are most needed. Importantly, TAGINE is agnostic to the specific taxonomic assignment used, allowing it to operate across different reference databases and taxonomic schemes without modification, and increasing its applicability even to settings beyond microbiome analysis.

TAGINE’s short runtime makes it a flexible and cost-effective processing step, which can be easily included in various data processing, analysis, or machine learning pipelines. Furthermore, its fast runtime on large datasets means that it does not necessitate aggressive filtering of rare taxa, which may be required prior to the application of other methods. This may prove crucial in datasets where this kind of prevalence or abundance filtering obscures the signal, ultimately degrading predictive performance. By using TAGINE, large initial feature sets can be retained without incurring significant runtime or performance costs.

Notably, despite these advantages, TAGINE has several limitations that warrant consideration. First, its speed is achieved, at least in part, at the expense of global optimality: the algorithm only explores the descendants of nodes that are expanded and never considers nodes whose parents do not increase information content. While a search for a globally optimal feature tree could potentially improve predictive performance, such an approach would likely incur a substantial increase in runtime, raise the risk of overfitting, and, based on our analyses, may not yield a significant gain in accuracy. Another limitation is that, as with all taxonomy-based methods, the results of the algorithm may be sensitive to tree misspecification and taxonomy assignment errors. Notably, however, such errors are more likely to occur at relatively fine-grained taxonomic resolution (e.g. genus or species) (Sczyrba *et al.* 2017, Poussin *et al.* 2022), rendering TAGINE (which initiates its search from the top of the tree) potentially more robust to such errors compared to other methods.

Importantly, each feature selected by TAGINE corresponds to a single clade in the taxonomic tree (without nesting across different levels), offering a highly interpretable feature set, especially compared to more domain-agnostic methods. In addition to selecting a set of features for downstream predictive modeling, this property of TAGINE also means that it yields a pruned taxonomic tree from which these features are derived. We propose that this pruned tree may, in and of itself, serve as a useful interpretative tool for exploring disease-specific microbial patterns. For instance, comparing the topologies of pruned trees across related versus unrelated diseases could reveal shared versus divergent microbial signatures. Moreover, examining which taxa are consistently retained or excluded across disease contexts may help identify species or clades that exhibit disease-specific variability.

## Supplementary Material

vbag056_Supplementary_Data

## Data Availability

The data underlying this article are available on GitHub (Curated Gut Microbiome Metabolome Data Resource), at https://dx.doi.org/10.1038/s41522-022-00345-5, and Zenodo (MicrobiomeHD: the human gut microbiome in health and disease), at https://dx.doi.org/10.5281/zenodo.1146764. The datasets were derived from sources in the public domain: https://github.com/borenstein-lab/microbiome-metabolome-curated-data; https://zenodo.org/records/1146764.
