# Supplementary material for: TAGINE: fast taxonomy-based feature engineering for microbiome analysis
Source: Bioinform Adv. 2026 Feb 17;6(1):vbag056. doi: 10.1093/bioadv/vbag056 (PMC12961271; doi:10.1093/bioadv/vbag056)
Supplement: vbag056_Supplementary_Data [file vbag056_supplementary_data.zip › Supplementary_Figures.pdf]

Supplementary Figures

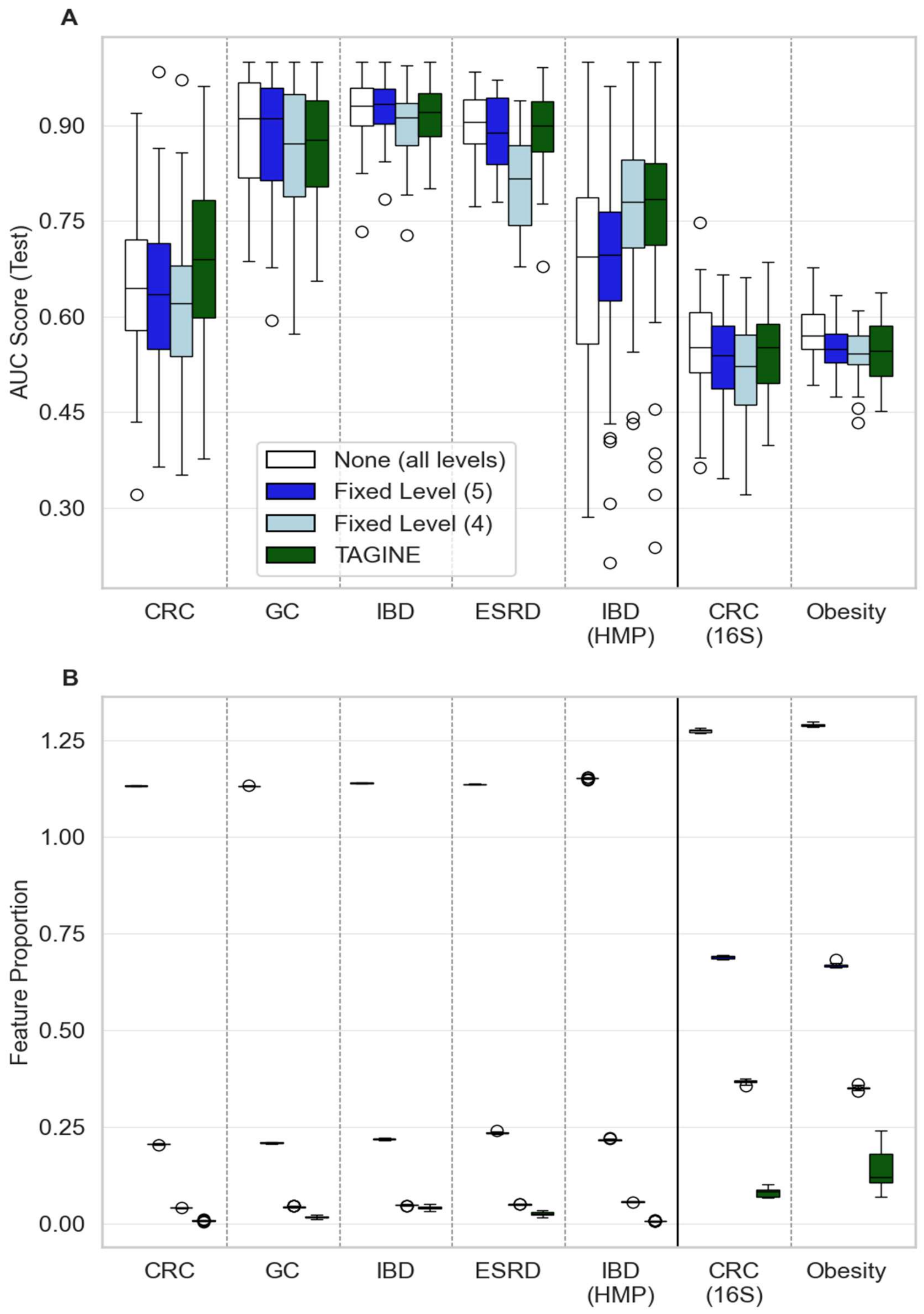

Supplementary Figure 1: Comparison of TAGINE's performance in terms of: **(A)** predictive accuracy, and **(B)** the proportion of features selected, relative to simple baselines that either use all features across *all* taxonomic levels (rather than only the finest-grained level used in the original feature set), or use all features at a fixed coarse-grained taxonomic level. Depths 4 and 5 correspond generally to the order and family levels, respectively.

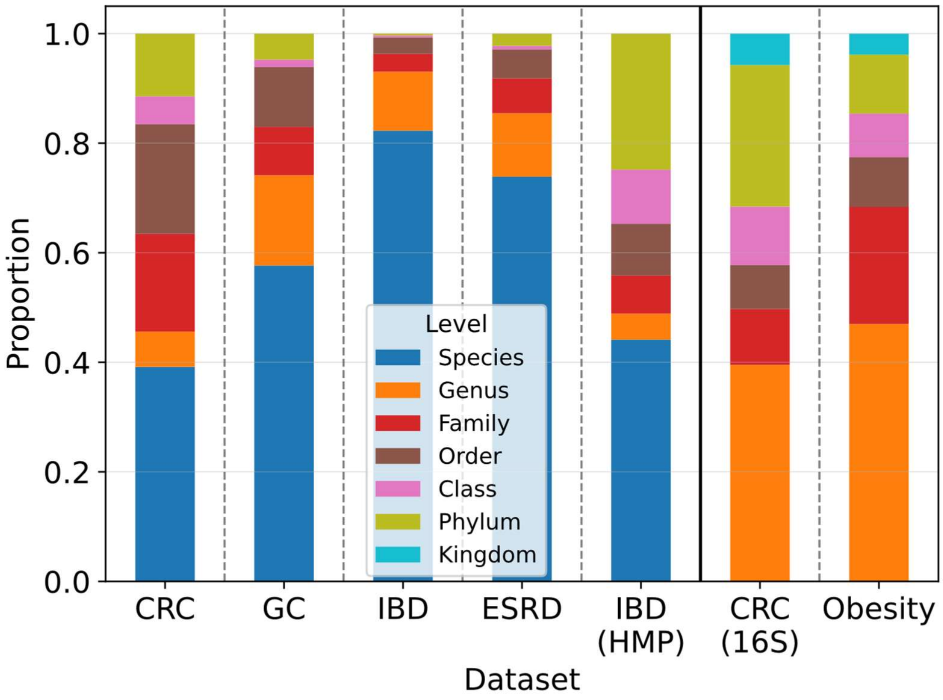

Supplementary Figure 2: Proportion of features selected by TAGINE at each taxonomic level per dataset.
