# Supplementary material for: TAGINE: fast taxonomy-based feature engineering for microbiome analysis
Source: Bioinform Adv. 2026 Feb 17;6(1):vbag056. doi: 10.1093/bioadv/vbag056 (PMC12961271; doi:10.1093/bioadv/vbag056)
Supplement: vbag056_Supplementary_Data [file vbag056_supplementary_data.zip › SuppTable1.pdf]

Dataset:	CRC			GC			IBD			ESRD			IBD (HMP)			CRC (16S)			Obesity		
# Features[a]:	6943.5			6824.0			7121.5			7081.3			5059.8			205.0			189.1		
	AUC[b] (ROC)	Prop[c] (Features)	Time[d] (s)	AUC[b] (ROC)	Prop[c] (Features)	Time[d] (s)	AUC[b] (ROC)	Prop[c] (Features)	Time[d] (s)	AUC[b] (ROC)	Prop[c] (Features)	Time[d] (s)	AUC[b] (ROC)	Prop[c] (Features)	Time[d] (s)	AUC[b] (ROC)	Prop[c] (Features)	Time[d] (s)	AUC[b] (ROC)	Prop[c] (Features)	Time[d] (s)
**TAGINE**	0.695 ± 0.130	0.008 ± 0.001	1.368 ± 0.125	0.872 ± 0.091	0.017 ± 0.003	1.802 ± 0.193	0.913 ± 0.051	0.041 ± 0.004	3.876 ± 0.338	0.893 ± 0.061	0.027 ± 0.004	2.770 ± 0.422	0.749 ± 0.162	0.007 ± 0.001	0.995 ± 0.282	0.545 ± 0.070	0.081 ± 0.009	0.397 ± 0.052	0.547 ± 0.047	0.137 ± 0.050	0.700 ± 0.128
**RFE (Scikit)**	0.576 ± 0.118	N/A	14.244 ± 0.553	0.893 ± 0.087	N/A	7.461 ± 0.284	0.923 ± 0.052	N/A	10.327 ± 0.588	0.929 ± 0.048	N/A	11.987 ± 0.507	0.643 ± 0.162	N/A	8.658 ± 2.716	0.566 ± 0.073	N/A	0.502 ± 0.029	0.568 ± 0.048	N/A	0.784 ± 0.135
**HFE (2018)**	0.730 ± 0.114	0.008 ± 0.003	148.960 ± 3.123	0.890 ± 0.083	0.090 ± 0.013	109.895 ± 1.478	0.926 ± 0.043	0.298 ± 0.027	326.006 ± 21.577	0.911 ± 0.044	0.140 ± 0.013	144.478 ± 7.706	-	-	-	0.523 ± 0.058	0.010 ± 0.005	14.117 ± 0.763	0.495 ± 0.045	0.014 ± 0.007	14.627 ± 0.827
**TaxaHFE (2023)**	0.648 ± 0.132	0.037 ± 0.017	1719.46 ± 43.096	0.881 ± 0.095	0.038 ± 0.007	711.688 ± 17.545	0.934 ± 0.044	0.046 ± 0.008	1281.79 ± 63.256	0.918 ± 0.051	0.047 ± 0.007	1589.7 ± 51.124	0.687 ± 0.164	0.058 ± 0.027	831.514 ± 35.202	0.573 ± 0.067	0.073 ± 0.035	211.617 ± 9.613	0.566 ± 0.045	0.114 ± 0.021	481.729 ± 15.964
**None**	0.639 ± 0.117	1.000 ± 0.000	N/A	0.891 ± 0.085	1.000 ± 0.000	N/A	0.915 ± 0.051	1.000 ± 0.000	N/A	0.901 ± 0.049	1.000 ± 0.000	N/A	0.678 ± 0.167	1.000 ± 0.000	N/A	0.545 ± 0.073	1.000 ± 0.000	N/A	0.575 ± 0.039	1.000 ± 0.000	N/A
**None (all levels)**	0.648 ± 0.128	1.133 ± 0.000	N/A	0.890 ± 0.094	1.132 ± 0.001	N/A	0.924 ± 0.050	1.140 ± 0.001	N/A	0.899 ± 0.053	1.137 ± 0.001	N/A	0.683 ± 0.158	1.152 ± 0.001	N/A	0.552 ± 0.079	1.275 ± 0.004	N/A	0.572 ± 0.040	1.290 ± 0.003	N/A
**Fixed Level (4)**	0.614 ± 0.111	0.041 ± 0.000	N/A	0.864 ± 0.102	0.044 ± 0.001	N/A	0.902 ± 0.055	0.049 ± 0.001	N/A	0.809 ± 0.072	0.050 ± 0.001	N/A	0.772 ± 0.126	0.057 ± 0.001	N/A	0.517 ± 0.073	0.367 ± 0.004	N/A	0.542 ± 0.039	0.351 ± 0.004	N/A
**Fixed Level (5)**	0.649 ± 0.128	0.206 ± 0.001	N/A	0.879 ± 0.100	0.209 ± 0.001	N/A	0.926 ± 0.045	0.219 ± 0.001	N/A	0.888 ± 0.057	0.235 ± 0.002	N/A	0.677 ± 0.146	0.217 ± 0.001	N/A	0.532 ± 0.074	0.689 ± 0.004	N/A	0.549 ± 0.040	0.668 ± 0.004	N/A

Supplementary Table 1: Comparison of AUC scores, proportion of features selected, and running time by dataset and selection method. Each cell notes the mean and standard deviation.

[a] The number of features originally included in each dataset (calculated as the mean across 50 train-test splits, each filtered independently for rare taxa).

[b] Mean Area Under the Curve (AUC) of the Receiver Operating Characteristic (ROC) curve.

[c] Mean proportion of features selected out of the original set of features.

[d] Mean runtime in seconds.
