# Supplementary material for: TAGINE: fast taxonomy-based feature engineering for microbiome analysis
Source: Bioinform Adv. 2026 Feb 17;6(1):vbag056. doi: 10.1093/bioadv/vbag056 (PMC12961271; doi:10.1093/bioadv/vbag056)
Supplement: vbag056_Supplementary_Data [file vbag056_supplementary_data.zip › SuppTable2.pdf]

Dataset:	HFE (2018) vs None	HFE(2018) vs RFE (Scikit)	HFE (2018) vs TAGINE	HFE (2018) vs TaxaHFE	None vs RFE (Scikit)	None vs TAGINE	None vs TaxaHFE	RFE (Scikit) vs TAGINE	RFE (Scikit) vs TaxaHFE	TAGINE vs TaxaHFE
**CRC**	0.0702	0.0702	0.421	0.0702	0.409	0.372	0.783	0.136	0.409	0.421
**GC**	0.984	0.984	0.984	0.984	0.984	0.984	0.984	0.984	0.984	0.984
**IBD**	0.818	0.877	0.818	0.818	0.818	0.895	0.694	0.818	0.818	0.818
**ESRD**	0.625	0.625	0.625	0.625	0.625	0.725	0.625	0.625	0.625	0.625
**IBD (HMP)**	-	-	-	-	0.804	0.772	0.834	0.772	0.772	0.772
**CRC (16S)**	0.726	0.726	0.726	0.726	0.726	0.992	0.726	0.726	0.919	0.726
**Obesity**	**0.0201**	**0.0201**	0.123	**0.0201**	0.819	0.4	0.764	0.552	0.902	0.552

Supplementary Table 2a: Multiple-testing-corrected p-values for AUC comparisons. Significance was evaluated using a Nadeau-Bengio-corrected two-sided t-test over the 50 train-test splits, with Benjamini-Hochberg FDR correction applied separately for each dataset. Significant results are in bold.

Dataset:	HFE (2018) vs TAGINE	HFE (2018) vs TaxaHFE	TAGINE vs TaxaHFE
**CRC**	0.873	**0.000451**	**0.000451**
**GC**	**5.69×10^{-16}**	**1.19×10^{-11}**	**7.82×10^{-08}**
**IBD**	**1.98×10^{-23}**	**2.14×10^{-22}**	0.229
**ESRD**	**1.28×10^{-25}**	**4.74×10^{-18}**	**5.72×10^{-07}**
**IBD (HMP)**	-	-	**5.67×10^{-05}**
**CRC (16S)**	**2.03×10^{-19}**	**0.00035**	0.583
**Obesity**	**1.59×10^{-06}**	**3.7×10^{-12}**	0.378

Supplementary Table 2b: Multiple-testing-corrected p-values for comparison of the proportion of selected features. Significance was evaluated using a Nadeau-Bengio-corrected two-sided t-test over the 50 train-test splits, with Benjamini-Hochberg FDR correction applied separately for each dataset. Significant results are in bold.

Dataset:	HFE (2018) vs RFE (Scikit)	HFE (2018) vs TAGINE	HFE (2018) vs TaxaHFE	RFE (Scikit) vs TAGINE	RFE (Scikit) vs TaxaHFE	TAGINE vs TaxaHFE
**CRC**	**6.75×10^{-57}**	**8.64×10^{-59}**	**6.5×10^{-54}**	**2.92×10^{-43}**	**9.25×10^{-56}**	**9.2×10^{-56}**
**GC**	**5.59×10^{-67}**	**1.47×10^{-68}**	**5.22×10^{-53}**	**4.38×10^{-36}**	**2.03×10^{-56}**	**1.92×10^{-56}**
**IBD**	**2.82×10^{-30}**	**1.89×10^{-30}**	**5.19×10^{-29}**	**5.25×10^{-24}**	**3.58×10^{-41}**	**3.58×10^{-41}**
**ESRD**	**1.02×10^{-37}**	**4.85×10^{-39}**	**2.03×10^{-48}**	**5.43×10^{-37}**	**3.2×10^{-50}**	**3.2×10^{-50}**
**IBD (HMP)**	-	-	-	**3.53×10^{-08}**	**2.9×10^{-45}**	**2.9×10^{-45}**
**CRC (16S)**	**4.06×10^{-39}**	**1.8×10^{-39}**	**6.08×10^{-42}**	**1.84×10^{-05}**	**4.04×10^{-43}**	**4.04×10^{-43}**
**Obesity**	**5.64×10^{-36}**	**7.93×10^{-36}**	**7.2×10^{-47}**	0.21	**1.17×10^{-49}**	**1.17×10^{-49}**

Supplementary Table 2c: Multiple-testing-corrected p-values for runtime comparisons. Significance was evaluated using a Nadeau-Bengio-corrected two-sided t-test over the 50 train-test splits, with Benjamini-Hochberg FDR correction applied separately for each dataset. Significant results are in bold.
